# Supplementary material for: Pex14p Phosphorylation Modulates Import of Citrate Synthase 2 Into Peroxisomes in Saccharomyces cerevisiae
Source: Front Cell Dev Biol. 2020 Sep 15;8:549451. doi: 10.3389/fcell.2020.549451 (PMC7522779; doi:10.3389/fcell.2020.549451)
Supplement: FIGURE S1 — Sequence coverage of Pex14p. [file Image_1.pdf]

```
  1 MSDVVSKDRK ALFDSAVSFL KDESIKDAPL LKKIEFLKSK GLTEKEIEIA
 51 MKEPKKDGIV GDEVSKKIGS TENRASQDMY LYEAMPPTLP HRDWKDYFVM
101 ATATAGLLYG AYEVTRRYVI PNILPEAKSK LEGDKKEIDD QFSKIDTVLN
151 AIEAEQAEFR KKESETLKEL SDTIAELKQA LVQTTRSREK IEDEFRIVKL
201 EVVNMQNTID KFVSDNDGMQ ELNNIQKEME SLKSLMNNRM ESGNAQDNRL
251 FSISPNGIPG IDTIPSASEI LAKMGMQEES DKEKENGSDA NKDDNAVPAW
301 KKAREQTIDS NASIPEWQKN TAANEISVPD WQNGQVEDSI PRTLQVDGSE
351 NLYFQ
```

Trypsin Asp-N Lys-C

Supplementary Figure S1: Sequence coverage of Pex14p. Pex14p was affinity-purified from crude membranes, proteolytically digested in solution with trypsin, Asp-N, or Lys-C, and subsequently analyzed by LC-MS. Tryptic peptides are shown in bold red, Asp-N peptides are underlined, and Lys-C peptides are highlighted in grey. Note that the sequence shown contains at the C-terminus the residue of the TPA tag (RTLQVDGSENLYFQ) that remained after TEV cleavages.
