# Supplementary material for: Pex14p Phosphorylation Modulates Import of Citrate Synthase 2 Into Peroxisomes in Saccharomyces cerevisiae
Source: Front Cell Dev Biol. 2020 Sep 15;8:549451. doi: 10.3389/fcell.2020.549451 (PMC7522779; doi:10.3389/fcell.2020.549451)
Supplement: FIGURE S2 — Annotated MS/MS spectra of Pex14p peptide ions comprising the phosphorylation site(s) indicated in the figure. [file Image_2.pdf]

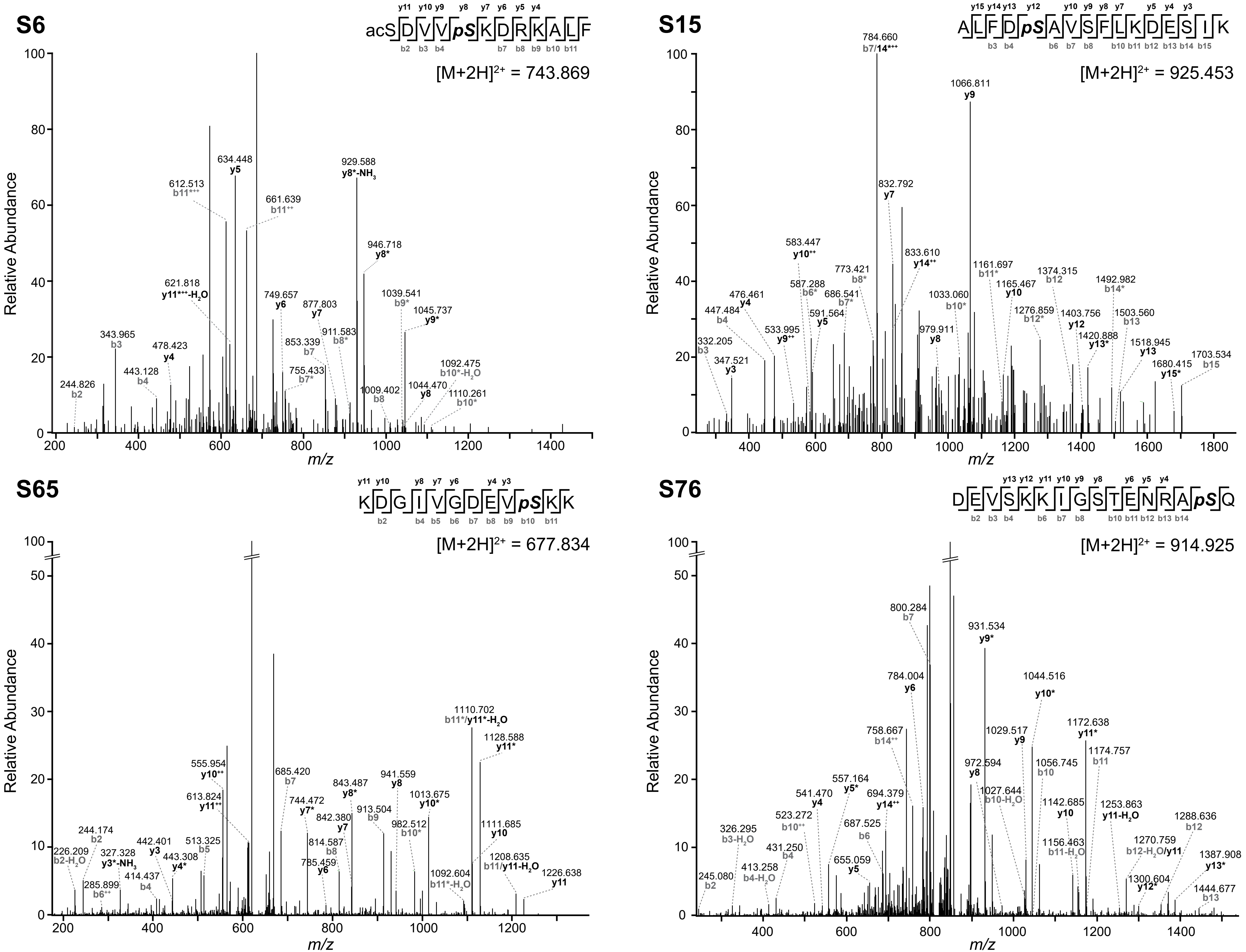

S6
acSDVVpSKDRKALF
[M+2H]2+ = 743.869
Relative Abundance
m/z
612.513 b11+++
634.448 y5
661.639 b11++
929.588 y8*-NH3
946.718 y8*
621.818 y11+++-H2O
749.657 y6
877.803 y7
1039.541 b9*
1045.737 y9*
343.965 b3
478.423 y4
443.128 b4
244.826 b2
853.339 b7
911.583 b8*
755.433 b7*
1092.475 b10*-H2O
1009.402 b8
1044.470 y8
1110.261 b10*
S15
ALFDpSAVSFLKDESIK
[M+2H]2+ = 925.453
784.660 b7/14*++
1066.811 y9
832.792 y7
583.447 y10++
833.610 y14++
773.421 b8*
587.288 b6*
686.541 b7*
476.461 y4
447.484 b4
533.995 y9++
591.564 y5
1161.697 b11*
1165.467 y10
1374.315 b12
1492.982 b14*
1033.060 b10*
1276.859 b12*
1403.756 y12
1503.560 b13
979.911 y8
1420.888 y13*
1518.945 y13
332.205 b3
347.521 y3
1680.415 y15*
1703.534 b15
S65
KDGIVGDEVpSKK
[M+2H]2+ = 677.834
1110.702 b11*/y11*-H2O
1128.588 y11*
555.954 y10++
685.420 b7
843.487 y8*
941.559 y8
613.824 y11++
744.472 y7*
913.504 b9
1013.675 y10*
842.380 y7
982.512 b10*
1111.685 y10
244.174 b2
814.587 b8
226.209 b2-H2O
327.328 y3*-NH3
442.401 y3
513.325 b5
785.459 y6
1208.635 b11/y11-H2O
443.308 y4*
414.437 b4
1092.604 b11*-H2O
1226.638 y11
285.899 b6++
S76
DEVSKKIGSTENRApSQ
[M+2H]2+ = 914.925
800.284 b7
931.534 y9*
784.004 y6
1044.516 y10*
1172.638 y11*
1029.517 y9
1174.757 b11
758.667 b14++
972.594 y8
1056.745 b10
557.164 y5*
694.379 y14++
1027.644 b10-H2O
541.470 y4
1253.863 y11-H2O
1288.636 b12
523.272 b10++
687.525 b6
1142.685 y10
326.295 b3-H2O
431.250 b4
1270.759 b12-H2O/y11
1156.463 b11-H2O
1387.908 y13*
655.059 y5
413.258 b4-H2O
245.080 b2
1300.604 y12*
1444.677 b13

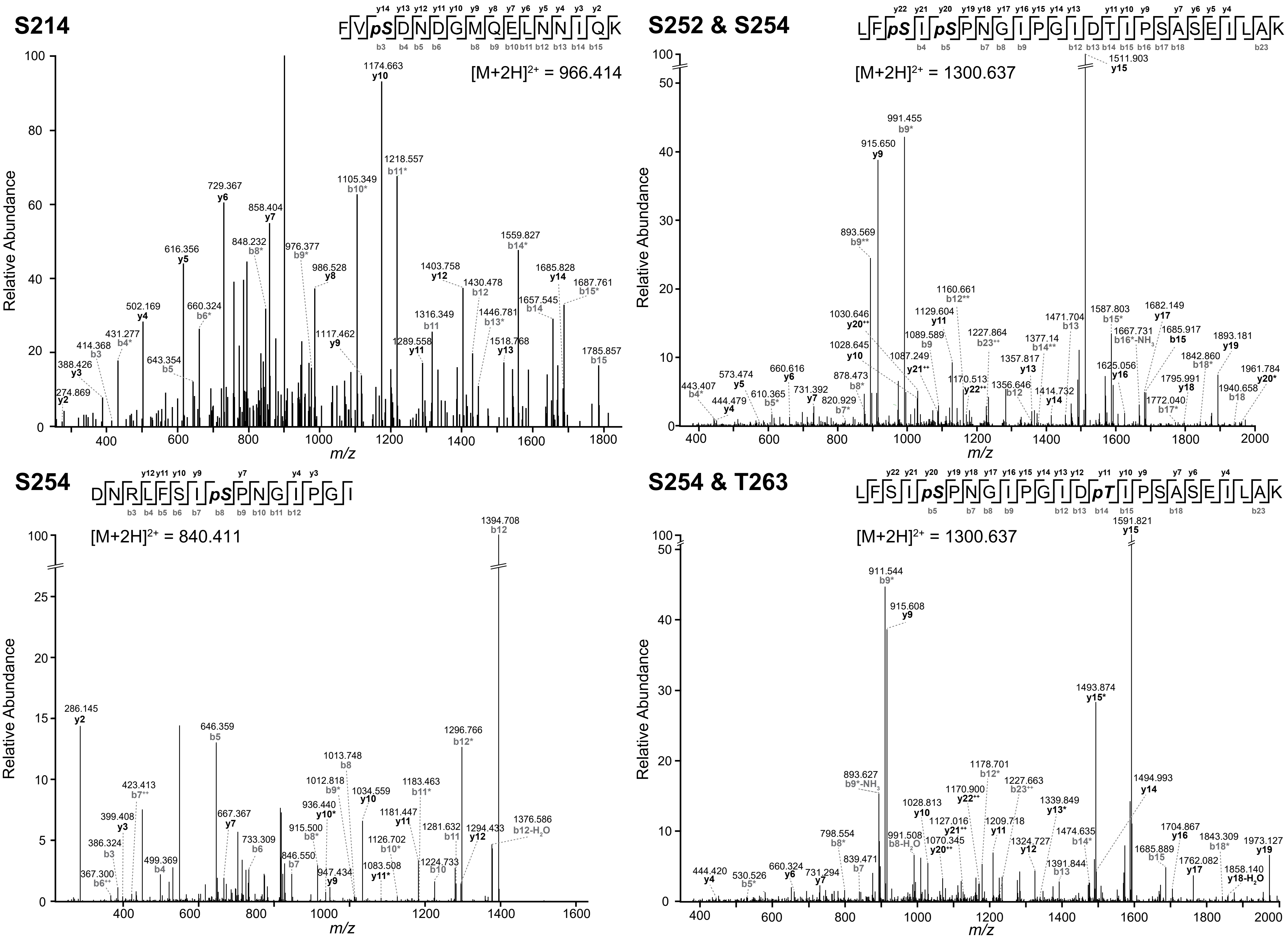
S214
FVpSDNDGMQELNNIQK
[M+2H]2+ = 966.414
1174.663 y10
1218.557 b11*
1105.349 b10*
729.367 y6
858.404 y7
848.232 b8*
976.377 b9*
986.528 y8
616.356 y5
1403.758 y12
1430.478 b12
1559.827 b14*
1685.828 y14
1687.761 b15*
1657.545 b14
1446.781 b13*
1316.349 b11
1289.558 y11
1518.768 y13
1117.462 y9
502.169 y4
660.324 b6*
431.277 b4*
414.368 b3
388.426 y3
643.354 b5
274.869 y2
1785.857 b15
Relative Abundance
m/z
S252 & S254
LFpSIpSPNGIPGIDTIPSASEILAK
[M+2H]2+ = 1300.637
1511.903 y15
991.455 b9*
915.650 y9
893.569 b9**
1160.661 b12**
1030.646 y20++
1129.604 y11
1089.589 b9
1028.645 y10
1087.249 y21++
1227.864 b23++
1170.513 y22++
1471.704 b13
1587.803 b15*
1682.149 y17
1667.731 b16*-NH3
1685.917 b15
1893.181 y19
1357.817 y13
1377.14 b14**
1356.646 b12
1414.732 y14
1625.056 y16
1842.860 b18*
1795.991 y18
1772.040 b17*
1940.658 b18
1961.784 y20*
443.407 b4*
573.474 y5
444.479 y4
610.365 b5*
660.616 y6
731.392 y7
878.473 b8*
820.929 b7*
S254
DNRLFSIpSPNGIPGI
[M+2H]2+ = 840.411
1394.708 b12
286.145 y2
646.359 b5
1296.766 b12*
1013.748 b8
1012.818 b9*
1034.559 y10
936.440 y10*
1183.463 b11*
1181.447 y11
423.413 b7++
399.408 y3
386.324 b3
367.300 b6++
499.369 b4
667.367 y7
733.309 b6
915.500 b8*
846.550 b7
947.434 y9
1126.702 b10*
1083.508 y11*
1224.733 b10
1281.632 b11
1294.433 y12
1376.586 b12-H2O
S254 & T263
LFSIpSPNGIPGIDpTIPSASEILAK
[M+2H]2+ = 1300.637
1591.821 y15
911.544 b9*
915.608 y9
1493.874 y15*
1494.993 y14
893.627 b9*-NH3
1178.701 b12*
1227.663 b23++
1170.900 y22++
1339.849 y13*
1028.813 y10
1127.016 y21++
1209.718 y11
991.508 b8-H2O
1070.345 y20++
1474.635 b14*
1324.727 y12
1391.844 b13
798.554 b8*
839.471 b7
1704.867 y16
1685.889 b15
1762.082 y17
1843.309 b18*
1858.140 y18-H2O
1973.127 y19
444.420 y4
530.526 b5*
660.324 y6
731.294 y7

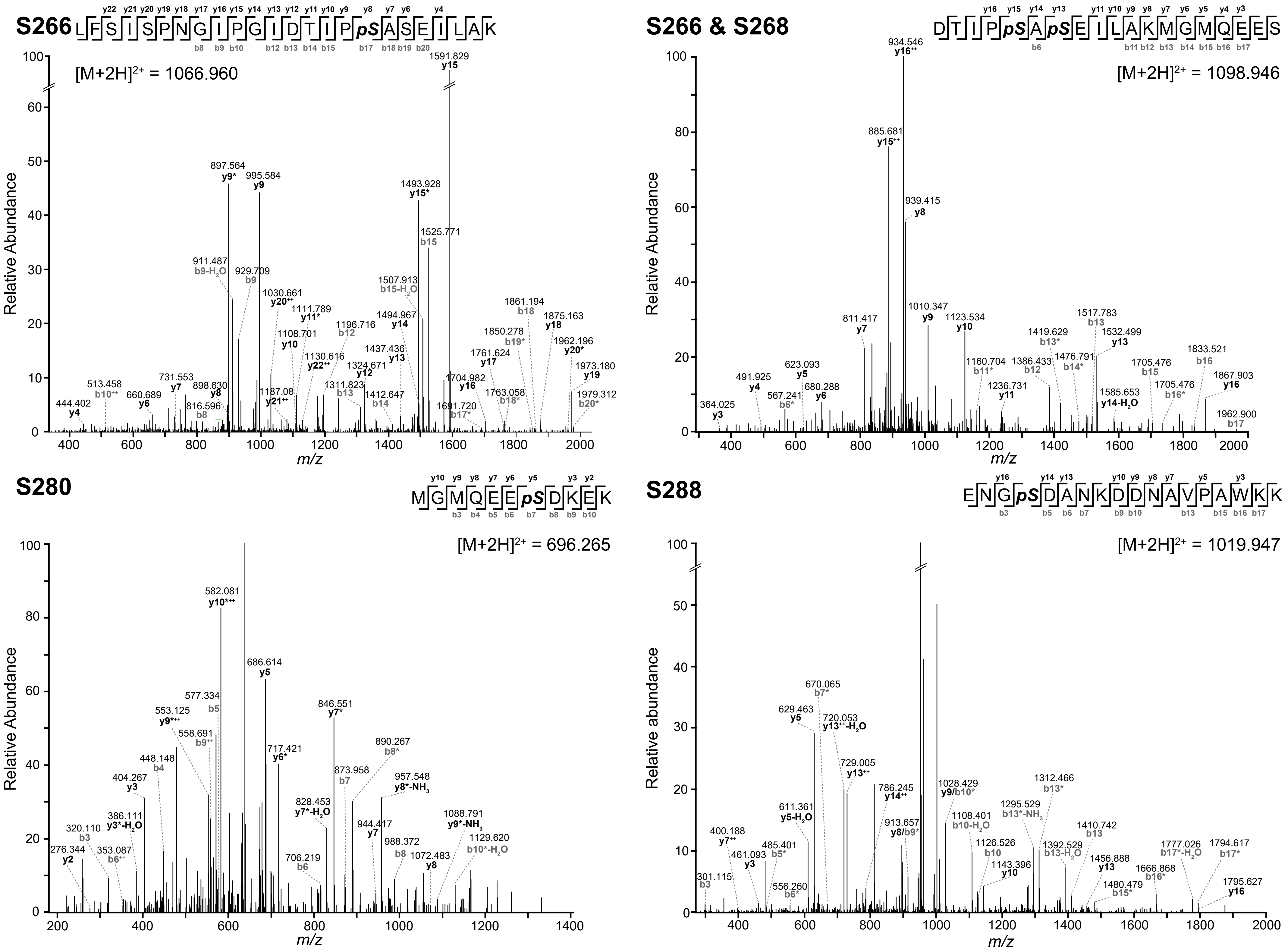

S266
LFSISPNGIPGIDTIPpSASEILAK
[M+2H]2+ = 1066.960
Relative Abundance
m/z
444.402 y4
513.458 b10++
660.689 y6
731.553 y7
816.596 b8
898.630 y8
897.564 y9*
911.487 b9-H2O
929.709 b9
995.584 y9
1030.661 y20++
1108.701 y10
1111.789 y11*
1130.616 y22++
1137.08 y21++
1196.716 b12
1311.823 b13
1324.671 y12
1412.647 b14
1437.436 y13
1493.928 y15*
1494.967 y14
1507.913 b15-H2O
1525.771 b15
1591.829 y15
1691.720 b17*
1704.982 y16
1761.624 y17
1763.058 b18*
1850.278 b19*
1861.194 b18
1875.163 y18
1962.196 y20*
1973.180 y19
1979.312 b20*
S266 & S268
DTIPpSApSEILAKMGMQEES
[M+2H]2+ = 1098.946
Relative Abundance
m/z
364.025 y3
491.925 y4
567.241 b6*
623.093 y5
680.288 y6
811.417 y7
885.681 y15++
934.546 y16++
939.415 y8
1010.347 y9
1123.534 y10
1160.704 b11*
1236.731 y11
1386.433 b12
1419.629 b13*
1476.791 b14*
1517.783 b13
1532.499 y13
1585.653 y14-H2O
1705.476 b15
1705.476 b16*
1833.521 b16
1867.903 y16
1962.900 b17
S280
MGMQEEpSDKEK
[M+2H]2+ = 696.265
Relative Abundance
m/z
276.344 y2
320.110 b3
353.087 b6++
386.111 y3*-H2O
404.267 y3
448.148 b4
553.125 y9*++
558.691 b9++
577.334 b5
582.081 y10*++
686.614 y5
706.219 b6
717.421 y6*
828.453 y7*-H2O
846.551 y7*
873.958 b7
890.267 b8*
944.417 y7
957.548 y8*-NH3
988.372 b8
1072.483 y8
1088.791 y9*-NH3
1129.620 b10*-H2O
S288
ENGpSDANKDDNAVPAWKK
[M+2H]2+ = 1019.947
Relative abundance
m/z
301.115 b3
400.188 y7++
461.093 y3
485.401 b5*
556.260 b6*
611.361 y5-H2O
629.463 y5
670.065 b7*
720.053 y13++-H2O
729.005 y13++
786.245 y14++
913.657 y8/b9*
1028.429 y9/b10*
1108.401 b10-H2O
1126.526 b10
1143.396 y10
1295.529 b13*-NH3
1312.466 b13*
1392.529 b13-H2O
1410.742 b13
1456.888 y13
1480.479 b15*
1666.868 b16*
1777.026 b17*-H2O
1794.617 b17*
1795.627 y16

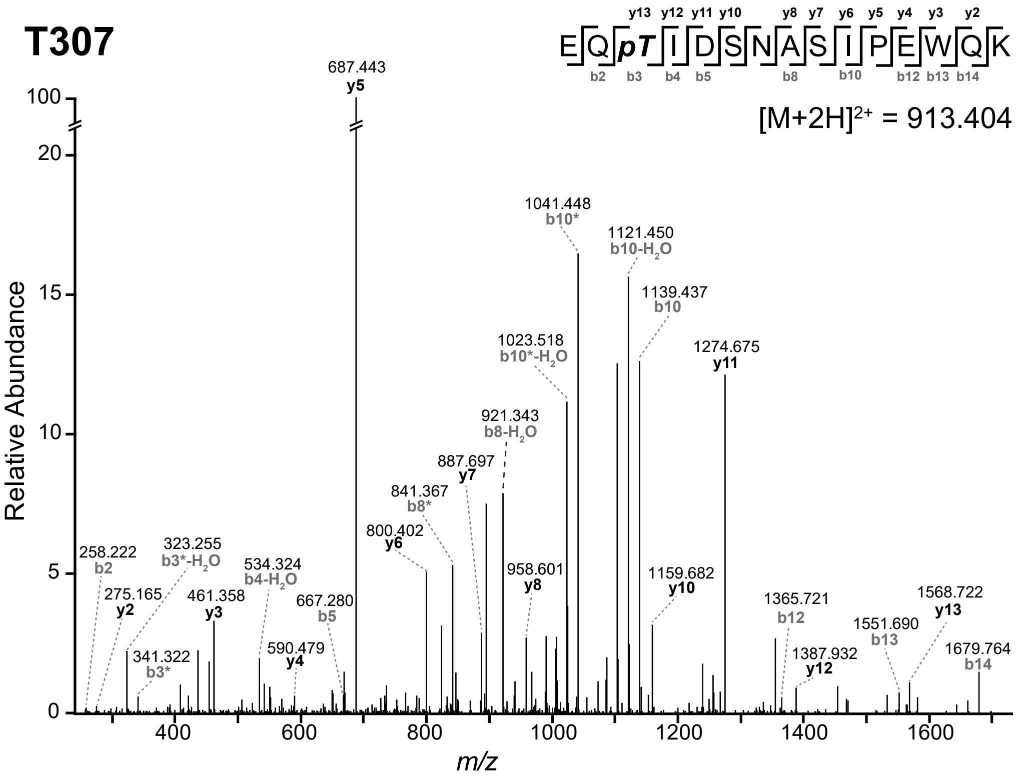

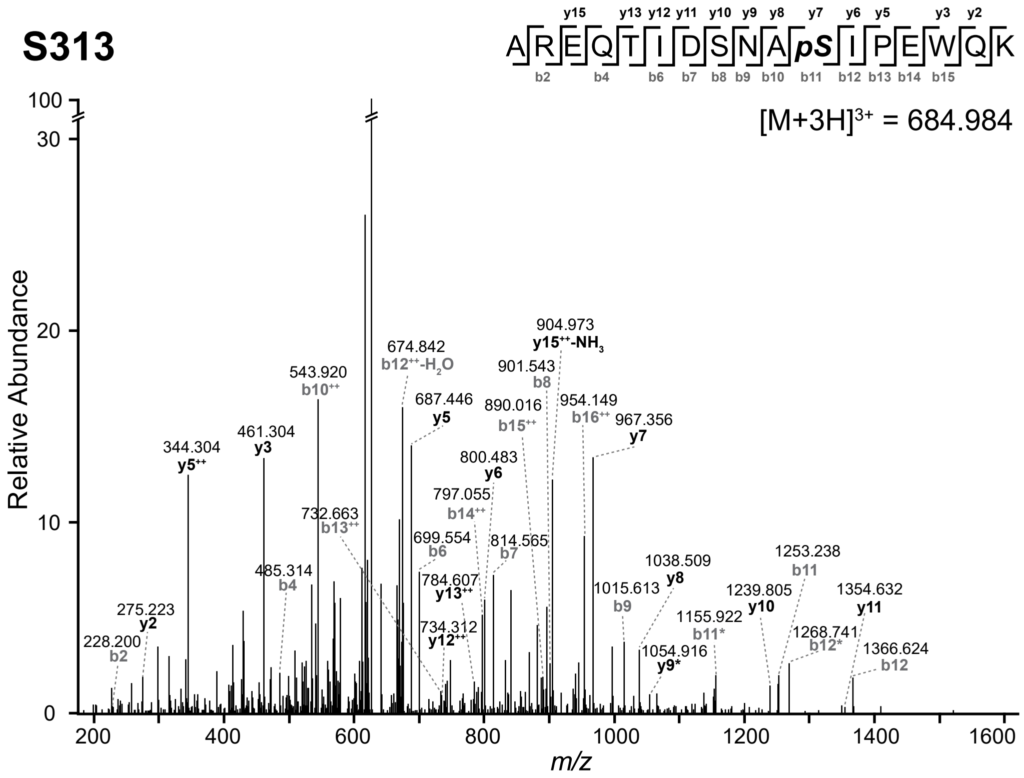

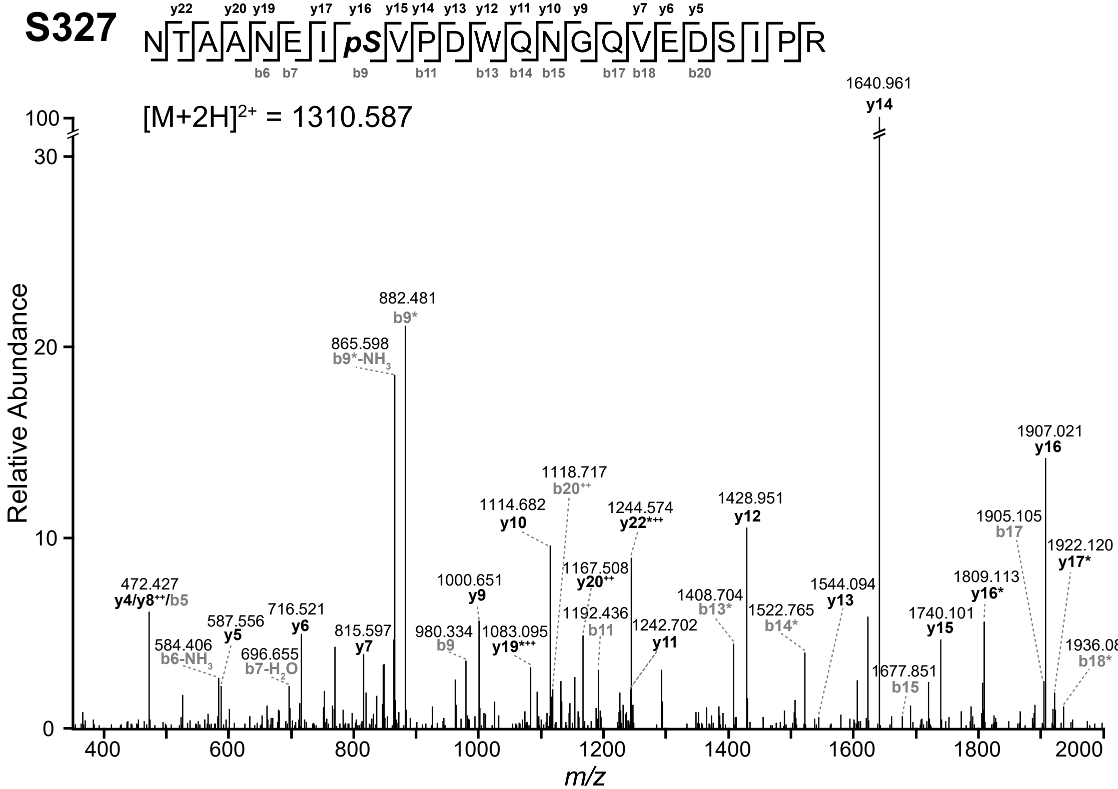

Supplementary Figure S2: Annotated MS/MS spectra of Pex14p peptide ions comprising the phosphorylation site(s) indicated in the figure. The spectra were acquired on an LTQ-Orbitrap XL using MSA for fragmentation of the precursor ion. ++, doubly protonated fragment ion; */** fragment ion with one/two neutral loss(es) of H_3PO_4; ac, acetylation.
