# Supplementary material for: Pex14p Phosphorylation Modulates Import of Citrate Synthase 2 Into Peroxisomes in Saccharomyces cerevisiae
Source: Front Cell Dev Biol. 2020 Sep 15;8:549451. doi: 10.3389/fcell.2020.549451 (PMC7522779; doi:10.3389/fcell.2020.549451)
Supplement: FIGURE S3 — Cells expressing GFP-SKL and Pex14pTPA wild-type, -S266A or -S266D exhibit comparable Pex14pTPA steady-state levels. [file Image_3.pdf]

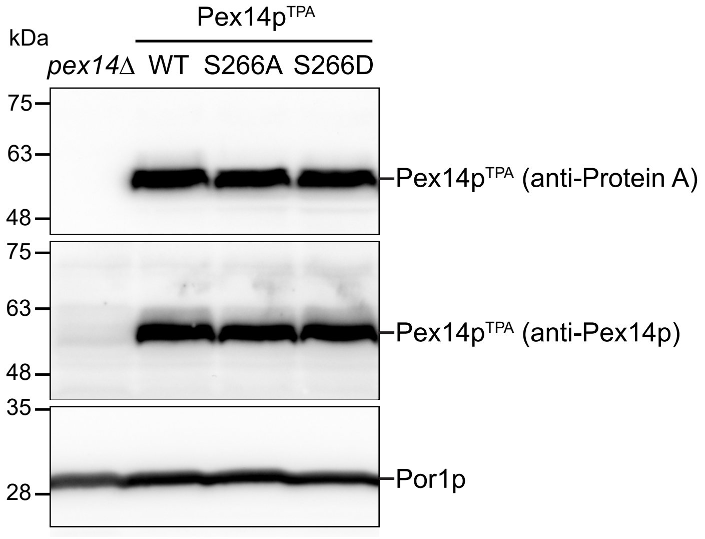

Supplementary Figure S3: Cells expressing GFP-SKL and Pex14p^{TPA} wild-type, S266A or S266D exhibit comparable Pex14p^{TPA} steady-state levels. Whole cell lysates obtained from cell expressing GFP-SKL and the indicated Pex14p^{TPA} variants or lacking *PEX14* (*pex14*Δ) were analyzed by immunoblotting using antisera recognizing Protein A, Pex14p, and the mitochondrial protein Por1p (loading control). WT, wild-type.
