# Supplementary material for: Pex14p Phosphorylation Modulates Import of Citrate Synthase 2 Into Peroxisomes in Saccharomyces cerevisiae
Source: Front Cell Dev Biol. 2020 Sep 15;8:549451. doi: 10.3389/fcell.2020.549451 (PMC7522779; doi:10.3389/fcell.2020.549451)
Supplement: FIGURE S4 — Fluorescence microscopy analysis of GFP-tagged peroxisomal matrix proteins in cells expressing Pex14pTPA wild-type, the Pex14p-S266A or -S266D mutant. [file Image_4.pdf]

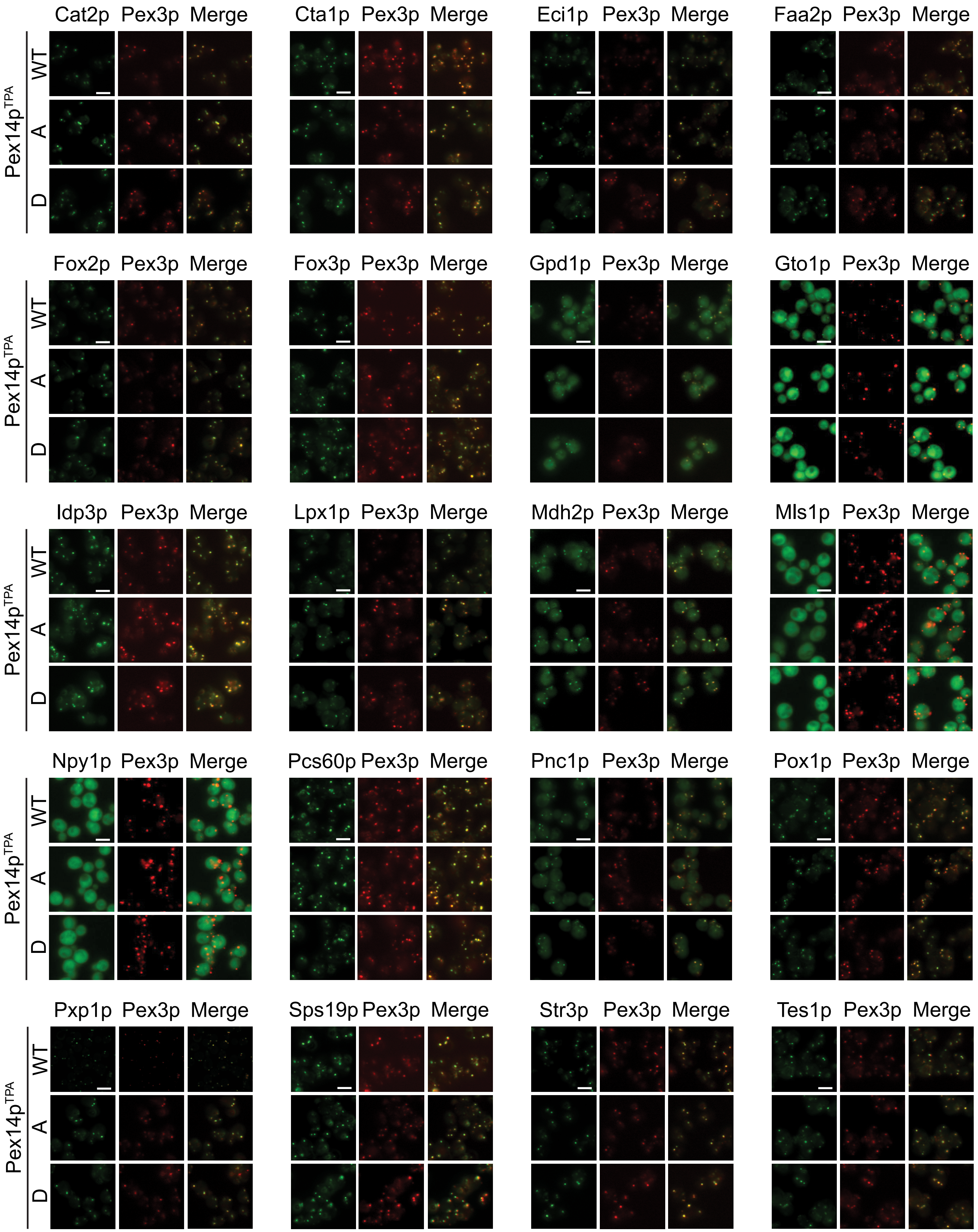

Supplementary Figure S4: Fluorescence microscopy analysis of GFP-tagged peroxisomal matrix proteins in cells expressing Pex14p^{TPA} wild-type, the S266A or S266D mutant. Same experiment as described for Figure 5A-C. Shown are representative images for the indicated proteins. WT, wild-type; A/D, S266A/D mutant. Scale bars, 5 µm.
