# Supplementary material for: Pex14p Phosphorylation Modulates Import of Citrate Synthase 2 Into Peroxisomes in Saccharomyces cerevisiae
Source: Front Cell Dev Biol. 2020 Sep 15;8:549451. doi: 10.3389/fcell.2020.549451 (PMC7522779; doi:10.3389/fcell.2020.549451)
Supplement: FIGURE S5 — Validation of data obtained in fluorescence microscopy experiments of GFP-Cit2p and GFP-Mdh3p cells. [file Image_5.pdf]

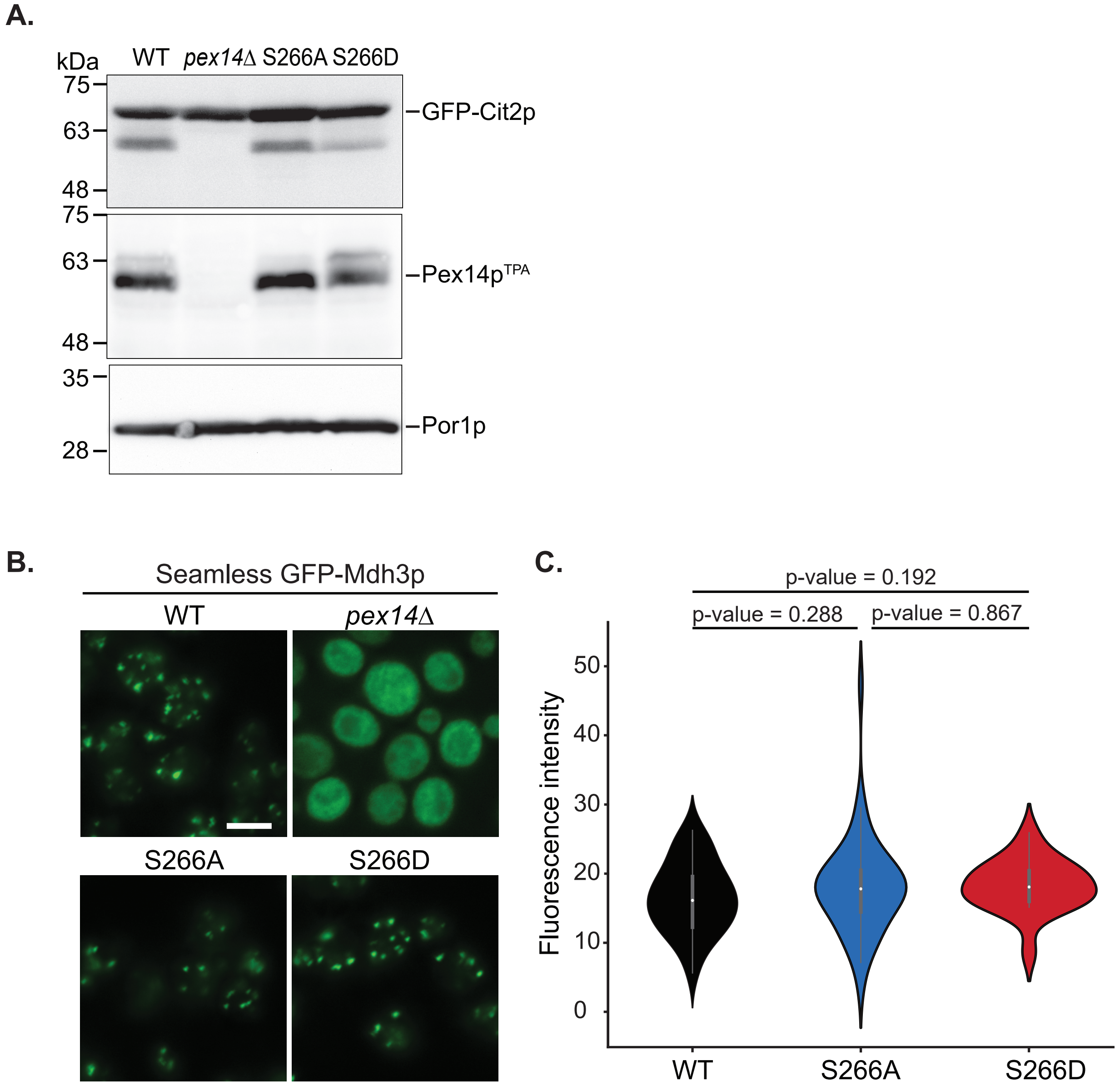

Supplementary Figure S5: Validation of data obtained in fluorescence microscopy experiments of GFP-Cit2p and GFP-Mdh3p cells. (**A**) Whole cell lysates prepared from cells of the same cultures that were used for the fluorescence microscopy analysis shown in Figure 5D were analyzed by immunoblotting using antisera recognizing GFP, Pex14p, and the mitochondrial protein Por1p, serving as loading control. WT, wild-type. (**B**) Same experiment as shown in Figure 5D using cells expressing the different Pex14p^{TPA} variants and seamless GFP-Mdh3p. Scale bar, 5 µm. (**C**) Quantification of the cytosolic fluorescence in seamless GFP-Mdh3p cells expressing Pex14p^{TPA}-wild-type, -S266A and -S266D grown in oleic acid as exemplarily shown in (B). p-values were calculated using the Welch's test (n = 33 for WT, n = 48 for S266A, n = 20 for S266D).
