# Supplementary material for: Pex14p Phosphorylation Modulates Import of Citrate Synthase 2 Into Peroxisomes in Saccharomyces cerevisiae
Source: Front Cell Dev Biol. 2020 Sep 15;8:549451. doi: 10.3389/fcell.2020.549451 (PMC7522779; doi:10.3389/fcell.2020.549451)
Supplement: FIGURE S6 — Increase of cellular Cit2p levels by overexpression of the CIT2 gene. [file Image_6.pdf]

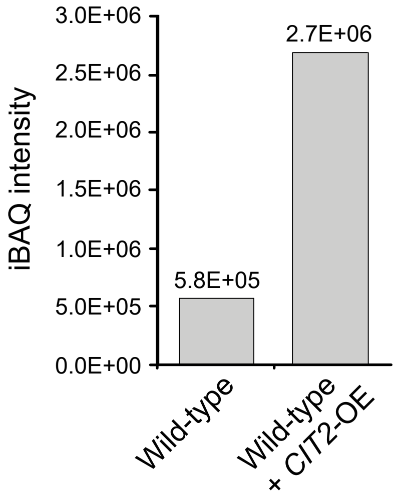

Supplementary Figure S6. Increase of cellular Cit2p levels by overexpression of the *CIT2* gene. Whole cell lysates of *S. cerevisiae* wild-type cells expressing endogenous Cit2p levels and wild-type cells transformed with a plasmid for overexpression of *CIT2* (*CIT2*-OE), grown in oleic acid as described in Figure 2A, were analyzed by LC-MS. Shown are iBAQ intensities as a measure of the cellular Cit2p abundance.
