# Supplementary material for: Pex14p Phosphorylation Modulates Import of Citrate Synthase 2 Into Peroxisomes in Saccharomyces cerevisiae
Source: Front Cell Dev Biol. 2020 Sep 15;8:549451. doi: 10.3389/fcell.2020.549451 (PMC7522779; doi:10.3389/fcell.2020.549451)
Supplement: FIGURE S7 — Growth of yeast cells expressing different Pex14pTPA variants in ethanol medium. [file Image_7.pdf]

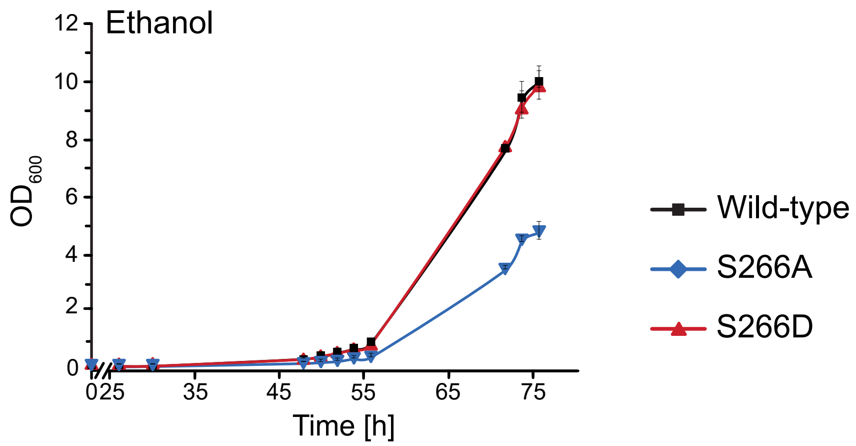

Supplementary Figure S7. Growth of yeast cells expressing different Pex14p^{TPA} variants in ethanol medium. Pex14p^{TPA} wild-type, -S266A or -S266D cells were pre-cultured in medium containing 0.3% glucose, grown for 16 h at 30°C and shifted to ethanol (2%) medium. Samples were taken at indicated time points and the OD_{600} was determined. Error bars indicate standard deviation (n = 3).
