# Supplementary material for: Pex14p Phosphorylation Modulates Import of Citrate Synthase 2 Into Peroxisomes in Saccharomyces cerevisiae
Source: Front Cell Dev Biol. 2020 Sep 15;8:549451. doi: 10.3389/fcell.2020.549451 (PMC7522779; doi:10.3389/fcell.2020.549451)
Supplement: TABLE S1 — Yeast strains used in this study. [file Table_1.pdf]

Supplementary Table S1. Yeast strains used in this study.

Strain	Genotype	Source or Reference	Primers	Template	Target Strain
Y04520	MATa; *ura3*Δ0; *leu2*Δ0; *his3*Δ1; *met15*Δ0; *pex14*::kanMX4	EUROSCARF, Giaever et al., 2002			
CB80	MATa, *ura3*-52, *leu2*-1, *trp1*-63, *his3*-200	Brocard et al., 1997			
CB199	CB80, *arg4*::natMX4, *lys1*::natMX3	Brocard et al., 1997			
SC03	CB80, *arg4*::natMX4, *lys1*::natMX3, *PEX14*:TPA-kanMX4	Oeljeklaus et al., 2012			
SC30	CB199, *pex14*:TPA-kanMX4	this study	015/018	Y04520	CB199
SC38	CB80, *arg4*::loxP, *lys1*::loxP	this study	O23/O24<br>O25/O26	pUG27<br>pUG73	CB80
SC73	CB80, *arg4*::loxP, *lys1*::loxP, *pex14*::kanMX4	this study	O15/O18	Y04520	SC38
SC260	CB80, *arg4*::natMX4, *lys1*::natMX3, *PEX14*::*URA3*	this study	RE4871/ RE4872	pUG35	CB80
SC271	CB80, *arg4*::natMX4, *lys1*::natMX3, *PEX14*^{S280A}:TPA-kanMX4	this study	O671/O674	pIS82	SC260
SC272	CB80, *arg4*::natMX4, *lys1*::natMX3, *PEX14*^{S280D}:TPA-kanMX4	this study	O671/O674	pIS83	SC260
SC274	CB80, *arg4*::natMX4, *lys1*::natMX3, *PEX14*^{S288A}:TPA-kanMX4	this study	O671/O674	pAS190	SC260
SC276	CB80, *arg4*::natMX4, *lys1*::natMX3, *PEX14*^{S288D}:TPA-kanMX4	this study	O671/O674	pAS209	SC260
SC305	CB80, *arg4*::natMX4, *lys1*::natMX3, *PEX14*^{S15A}:TPA-kanMX4	this study	O671/O674	pAS313	SC260
SC307	CB80, *arg4*::natMX4, *lys1*::natMX3, *PEX14*^{S6A}:TPA-kanMX4	this study	O671/O674	pAS309	SC260
SC310	CB80, *arg4*::natMX4, *lys1*::natMX3, *PEX14*^{S310A}:TPA-kanMX4	this study	O671/O674	pIS35	SC260
SC311	CB80, *arg4*::natMX4, *lys1*::natMX3, *PEX14*^{S310D}:TPA-kanMX4	this study	O671/O674	pIS36	SC260
SC312	CB80, *arg4*::natMX4, *lys1*::natMX3, *PEX14*^{S313A}:TPA-kanMX4	this study	O671/O674	pIS42	SC260
SC313	CB80, *arg4*::natMX4, *lys1*::natMX3, *PEX14*^{S313D}:TPA-kanMX4	this study	O671/O674	pIS43	SC260
SC314	CB80, *arg4*::natMX4, *lys1*::natMX3, *PEX14*^{T307A}:TPA-kanMX4	this study	O674/O675	pAS314	SC260
SC315	CB80, *arg4*::natMX4, *lys1*::natMX3, *PEX14*^{T307D}:TPA-kanMX4	this study	O671/O674	pAS372	SC260
SC319	CB80, *arg4*::natMX4, *lys1*::natMX3, *PEX14*^{S6D}:TPA-kanMX4	this study	O671/O674	pAS335	SC260
SC320	CB80, *arg4*::natMX4, *lys1*::natMX3, *PEX14*^{S15D}:TPA-kanMX4	this study	O671/O674	pAS325	SC260
SC321	CB80, *arg4*::natMX4, *lys1*::natMX3, *PEX14*^{S252A}:TPA-kanMX4	this study	O674/O675	pIS137	SC260

Strain	Genotype	Source or Reference	Primers	Template	Target Strain
SC322	CB80, *arg4*::natMX4, *lys1*::natMX3, *PEX14*^{S252D}:TPA-kanMX4	this study	O674/O675	pAS381	SC260
SC323	CB80, *arg4*::natMX4, *lys1*::natMX3, *PEX14*^{S254A}:TPA-kanMX4	this study	O674/O675	pIS80	SC260
SC324	CB80, *arg4*::natMX4, *lys1*::natMX3, *PEX14*^{S254D}:TPA-kanMX4	this study	O674/O675	pIS81	SC260
SC336	CB80, *arg4*::natMX4, *lys1*::natMX3, *PEX14*^{S65A}:TPA-kanMX4	this study	O674/O675	pIS78	SC260
SC337	CB80, *arg4*::natMX4, *lys1*::natMX3, *PEX14*^{S65D}:TPA-kanMX4	this study	O671/O674	pIS79	SC260
SC338	CB80, *arg4*::natMX4, *lys1*::natMX3, *PEX14*^{S76A}:TPA-kanMX4	this study	O671/O674	pAS310	SC260
SC339	CB80, *arg4*::natMX4, *lys1*::natMX3, *PEX14*^{S76D}:TPA-kanMX4	this study	O671/O674	pAS390	SC260
SC340	CB80, *arg4*::natMX4, *lys1*::natMX3, *PEX14*^{S214A}:TPA-kanMX4	this study	O671/O674	pAS252	SC260
SC341	CB80, *arg4*::natMX4, *lys1*::natMX3, *PEX14*^{S214D}:TPA-kanMX4	this study	O671/O674	pAS383	SC260
SC342	CB80, *arg4*::natMX4, *lys1*::natMX3, *PEX14*^{T263A}:TPA-kanMX4	this study	O674/O675	pAS392	SC260
SC343	CB80, *arg4*::natMX4, *lys1*::natMX3, *PEX14*^{T263D}:TPA-kanMX4	this study	O674/O675	pAS393	SC260
SC344	CB80, *arg4*::natMX4, *lys1*::natMX3, *PEX14*^{S266A}:TPA-kanMX4	this study	O674/O675	pAS384	SC260
SC345	CB80, *arg4*::natMX4, *lys1*::natMX3, *PEX14*^{S266D}:TPA-kanMX4	this study	O674/O675	pAS388	SC260
SC346	CB80, *arg4*::natMX4, *lys1*::natMX3, *PEX14*^{S268A}:TPA-kanMX4	this study	O674/O675	pAS382	SC260
SC347	CB80, *arg4*::natMX4, *lys1*::natMX3, *PEX14*^{S268D}:TPA-kanMX4	this study	O674/O675	pAS389	SC260
SC348	CB80, *arg4*::natMX4, *lys1*::natMX3, *PEX14*^{S327A}:TPA-kanMX4	this study	O674/O675	pAS311	SC260
SC349	CB80, *arg4*::natMX4, *lys1*::natMX3, *PEX14*^{S327D}:TPA-kanMX4	this study	O674/O675	pAS386	SC260
SC350*	CB80, *arg4*::natMX4, *lys1*::natMX3, *PEX14*$^{16S/T\rightarrow A}$:TPA-kanMX4	this study	O674/O675	pAS397	SC260
SC392*	CB80, *arg4*::natMX4, *lys1*::natMX3, *PEX14*$^{16S/T\rightarrow D}$:TPA-kanMX4	this study	O674/O675	pAS398	SC260
PCY2 Δ*pex14*	MATα, pex14::kanMX4, Δgal4, Δgal80, URA3::GAL1-lacZ, lys2-801amber, his3-Δ200, trp1-Δ63, leu2 ade2-101ochre	Huhse et al., 1998			
yMS 3438	MATa, *his3*Δ1, *leu2*Δ0, *lys2*Δ0, *ura3*Δ0, *met15*Δ0, *lys2+/lys*, *can1*Δ::STE2pr-spHIS5, *lyp1*Δ::STE3pr-LEU2, PEX3-mCherry::HIS, *pex14*Δ::NAT	this study			
SC371 (query strain)	MATa, *his3*Δ1, *leu2*Δ0, *lys2*Δ0, *ura3*Δ0, *met15*Δ0, *lys2+/lys*, *can1*Δ::STE2pr-spHIS5, *lyp1*Δ::STE3pr-LEU2, PEX3-mCherry::HIS, *pex14*::Pex14^{WT}:TPA-KanMX4	this study	O674/O675	pIS29	yMS 3438

Strain	Genotype	Source or Reference	Primers	Template	Target Strain
SC390 (query strain)	MATa, *his3*Δ1, *leu2*Δ0, *lys2*Δ0, *ura3*Δ0, *met15*Δ0, *lys2+/lys*, *can1*Δ::STE2pr-spHIS5, *lyp1*Δ::STE3pr-LEU2, PEX3-mCherry::HIS, *pex14*::Pex14^{S266A}:TPA-KanMX4	this study	O15/O18	SC344	yMS 3438
SC391 (query strain)	MATa, *his3*Δ1, *leu2*Δ0, *lys2*Δ0, *ura3*Δ0, *met15*Δ0, *lys2+/lys*, *can1*Δ::STE2pr-spHIS5, *lyp1*Δ::STE3pr-LEU2, PEX3-mCherry::HIS, *pex14*::Pex14^{S266D}:TPA-KanMX4	this study	O15/O18	SC345	yMS 3438
yMS4452	MATa; *his3*Δ1 *leu2*Δ0 *met15*Δ0 *ura3*Δ0, *GFP-CIT2*, *Δpex14*::kanMX4	this study			Seamlees GFP collection (Yofe et al. 2106)
yMS4453	MATa; *his3*Δ1 *leu2*Δ0 *met15*Δ0 *ura3*Δ0, GFP-Mdh3p, *Δpex14*::kanMX4	this study			Seamlees GFP collection (Yofe et al. 2106)
SC468	MATa; *his3*Δ1 *leu2*Δ0 *met15*Δ0 *ura3*Δ0, GFP-Cit2, *Δpex14*::NAT	this study	015/018	SC367	SC460
SC469	MATa; *his3*Δ1 *leu2*Δ0 *met15*Δ0 *ura3*Δ0, GFP-Mdh3, *Δpex14*::NAT	this study	015/018	SC367	SC461
SC502	MATa; *his3*Δ1 *leu2*Δ0 *met15*Δ0 *ura3*Δ0, GFP-Cit2, *pex14*::Pex14:TPA-KanMX4	this study	015/018	SC30	SC468
SC503	MATa; *his3*Δ1 *leu2*Δ0 *met15*Δ0 *ura3*Δ0, GFP-Cit2, *pex14*::Pex14^{S266A}:TPA-KanMX4	this study	015/018	SC344	SC468
SC504	MATa; *his3*Δ1 *leu2*Δ0 *met15*Δ0 *ura3*Δ0, GFP-Cit2, *pex14*::Pex14^{S266D}:TPA-KanMX4	this study	015/018	SC345	SC468
SC505	MATa; *his3*Δ1 *leu2*Δ0 *met15*Δ0 *ura3*Δ0, GFP-Mdh3, *pex14*::Pex14:TPA-KanMX4	this study	015/018	SC30	SC469
SC506	MATa; *his3*Δ1 *leu2*Δ0 *met15*Δ0 *ura3*Δ0, GFP-Mdh3, *pex14*::Pex14^{S266A}:TPA-KanMX4	this study	015/018	SC344	SC469
SC507	MATa; *his3*Δ1 *leu2*Δ0 *met15*Δ0 *ura3*Δ0, GFP-Mdh3, *pex14*::Pex14^{S266D}:TPA-KanMX4	this study	015/018	SC345	SC469

TPA, sequence coding for a cleavage site for the tobacco etch virus protease and Protein A.

*, Site mutations in the Pex14p 16S/T→A and D mutant are as follows: exchange of S6 , S15, S65, S76, S214, S252, S254, T263, S266, S268, S280, S288, T307, S310, S313, S327 to alanine or aspartate.

References Supplementary Table 1

Brocard, C., Lametschwandtner, G., Koudelka, R., and Hartig, A. (1997). Pex14p is a member of the protein linkage map of Pex5p. *EMBO J.* 16**,** 5491-5500. doi: 10.1093/emboj/16.18.5491

Giaever, G., Chu, A. M., Ni, L., Connelly, C., Riles, L., Veronneau, S., et al. (2002). Functional profiling of the *Saccharomyces cerevisiae* genome. *Nature* 418**,** 387-391. doi: 10.1038/nature00935

Huhse, B., Rehling, P., Albertini, M., Blank, L., Meller, K., and Kunau, W.-H. (1998). Pex17p of *Saccharomyces cerevisiae* is a novel peroxin and component of the peroxisomal protein translocation machinery. *J. Cell Biol.* 140**,** 49-60. doi: 10.1083/jcb.140.1.49

Oeljeklaus, S., Reinartz, B. S., Wolf, J., Wiese, S., Tonillo, J., Podwojski, K., et al. (2012). Identification of core components and transient interactors of the peroxisomal importomer by dual-track stable isotope labeling with amino acids in cell culture analysis. *J. Proteome Res.* 11**,** 2567-2580. doi: 10.1021/pr3000333
