# Supplementary material for: Pex14p Phosphorylation Modulates Import of Citrate Synthase 2 Into Peroxisomes in Saccharomyces cerevisiae
Source: Front Cell Dev Biol. 2020 Sep 15;8:549451. doi: 10.3389/fcell.2020.549451 (PMC7522779; doi:10.3389/fcell.2020.549451)
Supplement: TABLE S2 — Primers used in this study. [file Table_2.pdf]

Supplementary Table S2. Primers used in this study.

Primer	Sequence (5' to 3')
O5	GAACAAACTATTGATGCTAACGCCTCC
O6	GGAGGCGTTAGCATCAATAGTTTGTTC
O7	GAACAAACTATTGATGATAACGCCTCC
O8	GGAGGCGTTATCATCAATAGTTTGTTC
O15	GAATTTCAACAACGAATATACCTGC
O18	TTGCAGTTGTTGTTGTATCTTCTTC
O23	GAGCTCAAAAGCAGGTAACTATATAACAAGACTAAGGCAAACATGCAGCTGAAGCTTCGTACGC
O24	TACCAGACCTGATGAAATTCTTGCGCATAACGTCGCCATCTGCTAGCATAGGCCACTAGTGGATCTG
O25	AGATAACAACGAAAACGCTTTATTTTTCACACAACCGCAAAAATGCAGCTGAAGCTTCGTACGC
O26	TAAATGTCAGCGTAACGATAATGTATATACTTTAAATGTAAACTAGCATAGGCCACTAGTGGATCTG
O73	GCAACGCCGCTATTCCAGAATGGC
O74	GCCATTCTGGAATAGCGGCGTTGC
O75	GCAACGCCGATATTCCAGAATGGC
O76	GCCATTCTGGAATATCGGCGTTGC
O146	GAAAACGGCGCTGATGCTAATAAAG
O147	CTTTATTAGCATCAGCGCCGTTTTC
O148	GGCATGCAAGAAGAAGCTGATAAAGAAAAGG
O149	CCTTTTCTTTATCAGCTTCTTCTTGCATGCC
O150	CAGATTATTTTCCATAGCTCCTAATGGTATACCTGG
O151	CCAGGTATACCATTAGGAGCTATGGAAAATAATCTG
O188	GGCGATGAAGTAGCTAAAAAAATTGG
O189	CCAATTTTTTTAGCTACTTCATCGCC
O190	GGACAACAGATTATTTGCTATATCTCC
O191	GGAGATATAGCAAATAATCTGTTGTCC
O194	GATACGATTCCAGCTGCGTCTGAG
O195	CTCAGACGCAGCTGGAATCGTATC
O196	GAGAACAAGCTATTGATAGCAACG
O197	CGTTGCTATCAATAGCTTGTTCTC
O252	CTGCGGCTGAGATTCTTG
O253	CAAGAATCTCAGCCGCAG
O254	GAGAATAGAGCCGCTCAGGATATG
O255	CATATCCTGAGCGGCTCTATTCTC
O312	GCATCCTCGAGGTGGTCAATATGC
O321	GTACTGTCGACCTGCAGCGTAC
O325	CATTGTTCGACGCAGCAGTATC
O326	GATACTGCTGCGTCGAACAATG
O359	GAAAACGGCGACGATGCTAATAAAG
O360	CTTTATTAGCATCGTCGCCGTTTTC
O386	CGACAAATTTGTTGCAGATAATGACGGC
O387	GCCGTCATTATCTGCAACAAATTTGTCG
O388	CGACAAATTTGTTGATGATAATGACGGC
O389	GCCGTCATTATCATCAACAAATTTGTCG
O530	CGTGGTCGCTAAAGATCGTAAG
O531	CTTACGATCTTTAGCGACCACG
O532	CGTGGTCGATAAAGATCGTAAG

Primer	Sequence (5' to 3')
O533	CTTACGATCTTTATCGACCACG
O534	CATTGTTCGACGATGCAGTATC
O535	GATACTGCATCGTCGAACAATG
O536	GAGAATAGAGCCGATCAGGATATG
O537	CATATCCTGATCGGCTCTATTCTC
O538	GGACAACAGATTATTTGATATATCTCC
O539	GGAGATATATCAAATAATCTGTTGTCC
O540	GATACGATTCCAGATGCGTCTGAG
O541	CTCAGACGCATCTGGAATCGTATC
O542	CATCTGCGGATGAGATTCTTG
O543	CAAGAATCTCATCCGCAGATG
O544	GAGAACAAGATATTGATAGCAACG
O545	CGTTGCTATCAATATCTTGTTCTC
O546	CAATGAGATCGCTGTCCCTGAC
O547	GTCAGGGACAGCGATCTCATTG
O548	CAATGAGATCGATGTCCCTGAC
O549	GTCAGGGACATCGATCTCATTG
O550	CCTGGCATAGATGCGATTCCA
O551	TGGAATCGCATCTATGCCAGG
O552	CTGGCATAGATGATATTCCATCTG
O553	CAGATGGAATATCATCTATGCCAG
O554	CTGGCATAGATGATATTCCAGATG
O555	CATCTGGAATATCATCTATGCCAG
O671	GAAAACTCAAGTAAAACAGAGAAGTTGTAAGGTGAATAAGGAATGAGTGACGTGGTCAGTAAAG
O674	CAATTACAATTTCCGTTAAAAAACTAATTACTTACATAGAATTGCGGTTTAAACTGGATGGCGGCG
O675	GTTAAATTCTCTTTCACTAAAAACTTCTTTAAATAGCTCCAACTCTTCCCTCCCGCCATAATTGCAC
O1569	GAAAACGAGCTCGAATTCAAACGAGCTCATATATGGGGCCGTATACTT
O1570	TAACCCGGGGATCCGCATAAACAGATCTGGCGCGCCTTAATTAACCCGG
O1571	GGCGCGCCAGATCTGTTTATGCGGATCCCCGGGTTAATTAAGGCGCGCCAG
O1572	GCCCCATATATGAGCTCGTTTGAATTCGAGCTCGTTTTCGACACTGGATGGCG
O1620	GACGAGCTGTACAAGATGACAGTTCCTTATC
O1621	GCGGGCCTCGAGGCCCTATAGTTTGCTTTC
O1622	GAAAGCAAACTATAGGGCCTCGAGGCCCGC
O1623	GATAAGGAACTGTCATCTTGTACAGCTCGTC
OT1	GGCATGCAAGAAGAAGATGATAAAGAAAAGG
OT2	CCTTTTCTTTATCATCTTCTTCTTGCATGCC
OT3	GGCGATGAAGTAGATAAAAAAATTGG
OT4	CCAATTTTTTTATCTACTTCATCGCC
OT5	CAGATTATTTTCCATAGATCCTAATGGTATACCTGG
OT6	CCAGGTATACCATTAGGATCTATGGAAAATAATCTG
RE4871	TTTGAAAACTCAAGTAAAACAGAGAAGTTGTAAGGTGAATAAGGAACAGCTTTTCAATTCAATTCATCAT
RE4872	AATTACAATTTCCGTTAAAAAACTAATTACTTACATAGAATTGCGGGGTAATAACTGATATAATTAAATTG
RE6463	GCAAGAATCTCAGACGCAGCTGGAATCGTATCTATGC
RE6464	GCATAGATACGATTCCAGCTGCGTCTGAGATTCTTGC
KU107	GAATTCGAGGCCTTATGAGTGACGTGGTCAGT
RE6326	GATCGCGGCCGCctatgggatggagtcttc
