# Supplementary material for: Pex14p Phosphorylation Modulates Import of Citrate Synthase 2 Into Peroxisomes in Saccharomyces cerevisiae
Source: Front Cell Dev Biol. 2020 Sep 15;8:549451. doi: 10.3389/fcell.2020.549451 (PMC7522779; doi:10.3389/fcell.2020.549451)
Supplement: TABLE S3 — Plasmids and cloning strategies used in this study. [file Table_3.pdf]

Supplementary Table S3. Plasmids and cloning strategies used in this study.

Plasmid	Characteristics/Insert/ Comment	Backbone	Source or Reference	Primers	Restriction Enzyme	Template	Target Plasmid
pRS316	*CEN6, ARSH4, URA3, bla, lacZ*	pBlue-script KS	Sikorski and Hieter, 1989				
pRS416	*CEN6, ARSH4, URA3, bla, lacZ*	pBlue-script II	Sikorski and Hieter, 1989				
pUG27	*loxP*-Ag*TEF1*$_{Pro}$-Sp*HIS5*-Ag*TEF1*$_{Term}$-*loxP, bla*	pUG6	Güldener et al., 2002				
pUG35	*CEN6, ARSH4, URA3, MET25*$_{Pro}$*-EGFP-Cyc1*$_{Term}$	pRS416	Niedenthal et al., 1996				
pUG73	*loxP*-Kl*LEU2*$_{Pro}$-Kl*LEU2*-Ki*LEU2*$_{Term}$-*loxP, bla*	pUG6	Güldener et al., 2002				
pSH47	*CEN6, ARSH4, URA3, GAL1*$_{Pro}$*-Cre-CYC*$_{Term}$*, bla*	p416	Güldener et al., 1996				
pGFP-SKL	*Met25*$_{Pro}$*-GFP-SKL-Cyc1*$_{Term}$	pRS416	Schäfer et al., 2004				
pRS316-PEX14	*PEX14*$_{Pro}$*-PEX14-PEX14*$_{Term}$	pRS316	Albertini et al., 1997				
pIS24	*PEX14*$_{Pro}$*-PEX14-TPA*	pRS316	this study	O15/O18	EcoRI/XhoI	gDNA*	pRS316-PEX14
pIS29	*PEX14*$_{Pro}$*-PEX14-TPA*-ADH1$_{Term}$-*KanMX*	pRS316	this study	O15/O18	EcoRI	gDNA*	pIS24
pIS35	*PEX14*$_{Pro}$*-PEX14*^{S310A}*-TPA-ADH1*$_{Term}$*-KanMX*	pRS316	this study	O5/O6[a]		pIS29	pIS29
pIS36	*PEX14*$_{Pro}$*-PEX14*^{S310D}*-TPA-ADH1*$_{Term}$*-KanMX*	pRS316	this study	O7/O8[a]		pIS29	pIS29
pIS42	*PEX14*$_{Pro}$*-PEX14*^{S313A}*-TPA-ADH1*$_{Term}$*-KanMX*	pRS316	this study	O73/O74[a]		pIS29	pIS29
pIS43	*PEX14*$_{Pro}$*-PEX14*^{S313D}*-TPA-ADH1*$_{Term}$-KanMX	pRS316	this study	O75/O76[a]		pIS29	pIS29
pIS78	*PEX14*$_{Pro}$*-PEX14*^{S65A}*-TPA-ADH1*$_{Term}$*-KanMX*	pRS316	this study	O188/O189[a]		pIS29	pIS29
pIS79	*PEX14*$_{Pro}$*-PEX14*^{S65D}*-TPA-ADH1*$_{Term}$*-KanMX*	pRS316	this study	OT3/OT4[a]		pIS29	pIS29
pIS80	*PEX14*$_{Pro}$*-PEX14*^{S254A}*-TPA-ADH1*$_{Term}$*-KanMX*	pRS316	this study	O150/O151[a]		pIS29	pIS29
pIS81	*PEX14*$_{Pro}$*-PEX14*^{S254D}*-TPA-ADH1*$_{Term}$*-KanMX*	pRS316	this study	OT5/OT6[a]		pIS29	pIS29
pIS82	*PEX14*$_{Pro}$*-PEX14*^{S280A}*-TPA-ADH1*$_{Term}$*-KanMX*	pRS316	this study	O148/O149[a]		pIS29	pIS29
pIS83	*PEX14*$_{Pro}$*-PEX14*^{S280D}*-TPA-ADH1*$_{Term}$*-KanMX*	pRS316	this study	OT1/ OT2[a]		pIS29	pIS29
pIS137	*PEX14*$_{Pro}$*-PEX14*^{S252A}*-TPA-ADH1*$_{Term}$*-KanMX*	pRS316	this study	O190/O191[a]		pIS29	pIS29
pAS190	*PEX14*$_{Pro}$*-PEX14*^{S288A}*-TPA-ADH1*$_{Term}$*-KanMX*	pRS316	this study	O146/O147[a]		pIS29	pIS29

Plasmid	Characteristics/Insert/ Comment	Backbone	Source or Reference	Primers	Restriction Enzyme	Template	Target Plasmid
pAS209	$PEX14_{Pro}$-$PEX14^{S288D}$-*TPA*-$ADH1_{Term}$-*KanMX*	pRS316	this study	O359/O360[a]		pIS29	pIS29
pAS252	$PEX14_{Pro}$-$PEX14^{S214A}$-*TPA*-$ADH1_{Term}$-*KanMX*	pRS316	this study	O386/O387[a]		pIS29	pIS29
pAS309	$PEX14_{Pro}$-$PEX14^{S6A}$-*TPA*-$ADH1_{Term}$-*KanMX*	pRS316	this study	O530/O531[a]		pIS29	pIS29
pAS310	$PEX14_{Pro}$-$PEX14^{S76A}$-*TPA*-$ADH1_{Term}$-*KanMX*	pRS316	this study	O254/O255[a]		pIS29	pIS29
pAS311	$PEX14_{Pro}$-$PEX14^{S327A}$-*TPA*-$ADH1_{Term}$-*KanMX*	pRS316	this study	O546/O547[a]		pIS29	pIS29
pAS313	$PEX14_{Pro}$-$PEX14^{S15A}$-*TPA*-$ADH1_{Term}$-*KanMX*	pRS316	this study	O325/O326[a]		pIS29	pIS29
pAS314	$PEX14_{Pro}$-$PEX14^{T307A}$-*TPA*-$ADH1_{Term}$-*KanMX*	pRS316	this study	O196/O197[a]		pIS29	pIS29
pAS325	$PEX14_{Pro}$-$PEX14^{S15D}$-*TPA*-$ADH1_{Term}$-*KanMX*	pRS316	this study	O534/O535[a]		pIS29	pIS29
pAS335	$PEX14_{Pro}$-$PEX14^{S6D}$-*TPA*-$ADH1_{Term}$-*KanMX*	pRS316	this study	O532/O533[a]		pIS29	pIS29
pAS372	$PEX14_{Pro}$-$PEX14^{T307D}$-*TPA*-$ADH1_{Term}$-*KanMX*	pRS316	this study	O544/O545[a]		pIS29	pIS29
pAS381	$PEX14_{Pro}$-$PEX14^{S252D}$-*TPA*-$ADH1_{Term}$-*KanMX*	pRS316	this study	O538/O539[a]		pIS29	pIS29
pAS382	$PEX14_{Pro}$-$PEX14^{S268A}$-*TPA*-$ADH1_{Term}$-*KanMX*	pRS316	this study	O252/O253[a]		pIS29	pIS29
pAS383	$PEX14_{Pro}$-$PEX14^{S214D}$-*TPA*-$ADH1_{Term}$-*KanMX*	pRS316	this study	O388/O389[a]		pIS29	pIS29
pAS384	$PEX14_{Pro}$-$PEX14^{S266A}$-*TPA*-$ADH1_{Term}$-*KanMX*	pRS316	this study	O194/O195[a]		pIS29	pIS29
pAS386	$PEX14_{Pro}$-$PEX14^{S327D}$-*TPA*-$ADH1_{Term}$-*KanMX*	pRS316	this study	O548/O549[a]		pIS29	pIS29
pAS388	$PEX14_{Pro}$-$PEX14^{S266D}$-*TPA*-$ADH1_{Term}$-*KanMX*	pRS316	this study	O540/O541[a]		pIS29	pIS29
pAS389	$PEX14_{Pro}$-$PEX14^{S268D}$-*TPA*-$ADH1_{Term}$-*KanMX*	pRS316	this study	O542/O543[a]		pIS29	pIS29
pAS390	$PEX14_{Pro}$-$PEX14^{S76D}$-*TPA*-$ADH1_{Term}$-*KanMX*	pRS316	this study	O536/O537[a]		pIS29	pIS29
pAS392	$PEX14_{Pro}$-$PEX14^{T263A}$-*TPA*-$ADH1_{Term}$-*KanMX*	pRS316	this study	O550/O551[a]		pIS29	pIS29
pAS393	$PEX14_{Pro}$-$PEX14^{T263D}$-*TPA*-$ADH1_{Term}$-*KanMX*	pRS316	this study	O552/O553[a]		pIS29	pIS29
pAS335	$PEX14_{Pro}$-$PEX14^{S6D}$-*TPA*-$ADH1_{Term}$-*KanMX*	pRS316	this study	O532/O533[a]		pIS29	pIS29
pAS372	$PEX14_{Pro}$-$PEX14^{T307D}$-*TPA*-$ADH1_{Term}$-*KanMX*	pRS316	this study	O544/O545[a]		pIS29	pIS29
pAS381	$PEX14_{Pro}$-$PEX14^{S252D}$-*TPA*-$ADH1_{Term}$-*KanMX*	pRS316	this study	O538/O539[a]		pIS29	pIS29
pAS382	$PEX14_{Pro}$-$PEX14^{S268A}$-*TPA*-$ADH1_{Term}$-*KanMX*	pRS316	this study	O252/O253[a]		pIS29	pIS29

Plasmid	Characteristics/Insert/ Comment	Backbone	Source or Reference	Primers	Restriction Enzyme	Template	Target Plasmid
pAS383	*$PEX14_{Pro}$-$PEX14^{S214D}$-TPA-$ADH1_{Term}$-KanMX*	pRS316	this study	O388/O389[a]		pIS29	pIS29
pAS384	*$PEX14_{Pro}$-$PEX14^{S266A}$-TPA-$ADH1_{Term}$-KanMX*	pRS316	this study	O194/O195[a]		pIS29	pIS29
pAS386	*$PEX14_{Pro}$-$PEX14^{S327D}$-TPA-$ADH1_{Term}$-KanMX*	pRS316	this study	O548/O549[a]		pIS29	pIS29
pAS388	*$PEX14_{Pro}$-$PEX14^{S266D}$-TPA-$ADH1_{Term}$-KanMX*	pRS316	this study	O540/O541[a]		pIS29	pIS29
pAS389	*$PEX14_{Pro}$-$PEX14^{S268D}$-TPA-$ADH1_{Term}$-KanMX*	pRS316	this study	O542/O543[a]		pIS29	pIS29
pAS390	*$PEX14_{Pro}$-$PEX14^{S76D}$-TPA-$ADH1_{Term}$-KanMX*	pRS316	this study	O536/O537[a]		pIS29	pIS29
pAS392	*$PEX14_{Pro}$-$PEX14^{T263A}$-TPA-$ADH1_{Term}$-KanMX*	pRS316	this study	O550/O551[a]		pIS29	pIS29
pAS393	*$PEX14_{Pro}$-$PEX14^{T263D}$-TPA-$ADH1_{Term}$-KanMX*	pRS316	this study	O552/O553[a]		pIS29	pIS29
pAS397[b,c]	*$PEX14_{Pro}$-$PEX14^{16A}$- TPA-$ADH1_{Term}$-KanMX*	pRS316	this study	O188/O189[a]<br>O254/O255[a]<br>O325/O326[a]<br>O386/O387[a]<br>O530/O531[a]<br>O546/O547[a]<br>O550/O551[a]<br>O312/O321	XhoI/SalI	pIS29,<br>GeneArt string[d]	pIS29
pAS398[b,e]	*$PEX14_{Pro}$-$PEX14^{16D}$-TPA-$ADH1_{Term}$-KanMX*	pRS316	this study	O388/O389[a]<br>O532/O533[a]<br>O548/O549[a]<br>O554/O555[a]	XbaI/SalI	pEX-K-PEX14[f]	pIS29
pEX-K-PEX14[f]	*Kan*, pUC ori, *$PEX14_{Pro}$-$PEX14^{12D}$*		Eurofins, this study			Gene synthesis	
pPC86	*$ADC1_{PRO}$-GAL4-activation domain* (*Gal4-AD*)		Chevray and Nathans, 1992				
pPC97	*$ADC1_{PRO}$-GAL4-DNA binding domain* (*Gal4-BD*)		Chevray and Nathans, 1992				
pPC86 + PEX5	*$ADC1_{PRO}$-Gal4-AD-PEX5*	pPC86	Erdmann and Blobel, 1996				
pPC97 + PEX13	*$ADC1_{PRO}$-Gal4-BD-PEX13*	pPC97	Girzalsky et al., 1999				
pPC86 + PEX14	*$ADC1_{PRO}$-Gal4-AD-PEX14*	pPC86	Albertini et al., 1997				
pPC97 + PEX14	*$ADC1_{PRO}$-Gal4-BD-PEX14*	pPC97	Albertini et al., 1997				

Plasmid	Characteristics/Insert/ Comment	Backbone	Source or Reference	Primers	Restriction Enzyme	Template	Target Plasmid
pPC97 + PEX17	*ADC1*$_{PRO}$*-Gal4-BD-PEX17*	pPC97	Albertini et al., 1997				
pPC86 + PEX14^{S266D}	*ADC1*$_{PRO}$*-Gal4-AD-PEX14*^{S266D}	pPC86	this study	KU107/ RE6326	EcoRI/NotI	gDNA	
pPC86 + PEX14^{S266A}	*ADC1*$_{PRO}$*-Gal4-AD-PEX14*^{S266A}	pPC86	this study	RE6463/ RE6464		pPC86 + PEX14	
pPC97 + PEX14^{S266D}	*ADC1*$_{PRO}$*-Gal4-BD-PEX14*^{S266D}	pPC97	this study		SalI/NotI	pPC86 + PEX14^{S266D}	
pPC97 + PEX14^{S266A}	*ADC1*$_{PRO}$*-Gal4-BD-PEX14*^{S266A}	pPC97	this study	RE6463/ RE6464		pPC97 + PEX14	
N'TEF2 mCherry	*URA:: TEF2*$_{PRO}$*-mCherry*	pFA6	Breslow et al., 2008				
pRM596	*NAT::TEF2*$_{PRO}$*-mCherry*	pFA6	this study	01569+01570 01571/01572[g]		gDNA**	N'TEF2 mCherry
pRM613	*NAT::TEF2*$_{PRO}$*-mCherry-CIT2*	pFA6	this study	01620/01621 01622/01623[g]		gDNA*	pRM596

Pro, promoter; Term, terminator; TPA, sequence coding for a cleavage site for the tobacco etch virus protease and Protein A

* Genomic DNA (gDNA) was prepared from SC03

** Genomic DNA (gDNA) was prepared from yMS 3438

[a], Used for site-directed mutagenesis according to Papworth *et al.* (1996).

[b], Site mutations in the Pex14p-16A and -16D mutants are as follows: exchange of S6, S15, S65, S76, S214, S252, S254, T263, S266, S268, S280, S288, T307, S310, S313, S327 to alanine (16A) or aspartate (16D).

[c], For generation of the Pex14p-16A mutant, S6 (O530/O531), S15 (O325/O326), S65 (O188/O189), S76 (O254/O255), S214 (O386/O387), T263 (O550/O551), and S327 (O546/O547) to Ala mutations were introduced by consecutive site-directed mutageneses. The remaining 9 site mutations were introduced using gene synthesis (GeneArt Strings DNA Fragments, Thermo Fisher Scientific), amplified by PCR (O312/O321) and inserted in XhoI/SalI sites.

[d], The GeneArt String DNA fragment (Thermo Fisher Scientific) comprises the region between nucleotides 604 and 1023 of *PEX14* including codon changes resulting in the indicated S/T-to-A (GCT) exchanges and flanking sequences with restriction sites for XhoI at the 5' (gcatcctcgag) and for SalI at the 3' end (cgtacgctgcaggtcgacagtac).

[e], For generation of the Pex14p-16D mutant, S6 (O532/O533), S214 (O388/O389), T263 (O554/O555), and S327 (O548/O549) to Asp mutations were introduced by consecutive site-directed mutageneses. The remaining 12 site mutations were introduced using gene synthesis (Eurofins), integrated in the vector pEX-K and ligated in the target vector in XbaI/SalI sites.

[f], The plasmid comprises the sequence of the *PEX14* promoter region (614 nts upstream) and the PEX14 ORF with exchange of the codons for the serine/threonine residues of S15, S65, S76, S252, S254, S266, S268, S280, S288, T307, S310, S313 to aspartate (GAT) and flanking sequences with restriction sites for XbaI at the 5' (tctagaactagtggatcc) and for SalI at the 3' end (cgtacgctgcaggtcgac).

[g], Primers were used for FastCloning (Li et al., 2011)

References Supplementary Table 3

Albertini, M., Rehling, P., Erdmann, R., Girzalsky, W., Kiel, J. A. K. W., Veenhuis, M., et al. (1997). Pex14p, a peroxisomal membrane protein binding both receptors of the two PTS-dependent import pathways. *Cell* 89**,** 83-92. doi: 10.1016/S0092-8674(00)80185-3

Breslow, D.K., Cameron, D. M., Collins, S. R., Schuldiner, M., Stewart-Ornstein, J., Newman, H. W., et al. (2008). A comprehensive strategy enabling high-resolution functional analysis of the yeast genome. *Nat. Methods* 5, 711-718, doi: 10.1038/nmeth.1234

Chevray, P. M., and Nathans, D. (1992). Protein interaction cloning in yeast: identification of mammalian proteins that react with the leucine zipper of Jun. *Proc. Natl. Acad. Sci. USA* 89**,** 5789-5793. doi: 10.1073/pnas.89.13.5789

Erdmann, R., and Blobel, G. (1996). Identification of Pex13p a peroxisomal membrane receptor for the PTS1 recognition factor. *J. Cell Biol.* 135**,** 111-121. doi: 10.1083/jcb.135.1.111

Girzalsky, W., Rehling, P., Stein, K., Kipper, J., Blank, L., Kunau, W. H., et al. (1999). Involvement of Pex13p in Pex14p localization and peroxisomal targeting signal 2-dependent protein import into peroxisomes. *J. Cell Biol.* 144**,** 1151-1162. doi: 10.1083/jcb.144.6.1151

Güldener, U., Heck, S., Fielder, T., Beinhauer, J., and Hegemann, J. H. (1996). A new efficient gene disruption cassette for repeated use in budding yeast. *Nucleic Acids Res.* 24**,** 2519-2524. doi: 10.1093/nar/24.13.2519

Güldener, U., Heinisch, J., Köhler, G. J., Voss, D., and Hegemann, J. H. (2002). A second set of loxP marker cassettes for Cre-mediated multiple gene knockouts in budding yeast. *Nucleic Acids Res.* 30**,** e23. doi: 10.1093/nar/30.6.e23

Li, C., Wen, A., Shen, B., Lu, J., Huang, Y., and Chang, Y. (2011). FastCloning: a highly simplified, purification-free, sequence- and ligation-independent PCR cloning method. *BMC Biotechnology* 11, doi: 10.1186/1472-6750-11-92

Niedenthal, R. K., Riles, L., Johnston, M., and Hegemann, J. H. (1996). Green fluorescent protein as a marker for gene expression and subcellular localization in budding yeast. *Yeast* 12**,** 773-786. doi: 10.1002/(SICI)1097-0061(19960630)12:8%3C773::AID-YEA972%3E3.0.CO;2-L

Papworth, C., Bauer, J. C., and Braman, J. C.(1996). Site-directed mutagenesis in one day with >80% efficiency. *Strategies* 9**,** 3-4.

Schäfer, A., Kerssen, D., Veenhuis, M., Kunau, W. H., and Schliebs, W. (2004). Functional similarity between the peroxisomal PTS2 receptor binding protein Pex18p and the N-terminal half of the PTS1 receptor Pex5p. *Mol. Cell. Biol.* 24**,** 8895-8906. doi: 10.1128/MCB.24.20.8895-8906.2004

Sikorski, R. S., and Hieter, P. (1989). A system of shuttle vectors and yeast host strains designed for efficient manipulation of DNA in *Saccharomyces cerevisiae*. *Genetics* 122**,** 19-27.
